# Plasma lncRNA FEZF1-AS1 as a potential biomarker for diagnosis of non-small-cell lung carcinoma

**DOI:** 10.1097/MD.0000000000021019

**Published:** 2020-06-26

**Authors:** Yajie Huang, Guangjie Liu, Handie Ma, Yanpeng Tian, Changjie Huang, Fang Liu, Yuxuan Jia, Da Jiang

**Affiliations:** a Department of Medical Oncology, The Fourth Hospital of Hebei Medical University; b Undergraduate of Clinical Medicine, Hebei Medical University; c Department of Thoracic Surgery; d Department of Radiotherapy, The Fourth Hospital of Hebei Medical University; e Department of Obstetrics and Gynecology, the Second Hospital of Hebei Medical University, Shijiazhuang, Hebei; f Undergraduate of Clinical Medicine, Southern Medical University, Guangzhou, Guangdong, China.

**Keywords:** biomarker, long non-coding RNA, non-small-cell lung carcinoma, plasma

## Abstract

Diagnosis of numerous cancers has been closely linked to the expression of certain long non-coding RNAs. This study aimed to evaluate levels of plasma FEZ family zinc finger 1 antisense RNA 1 (FEZF1-AS1) relative to non-small-cell lung carcinoma (NSCLC) diagnosis.

The level of FEZF1-AS1 in the blood plasma of 126 NSCLC patients and 62 healthy controls was examined by quantitative real-time polymerase chain reaction.

Plasma FEZF1-AS1 of the NSCLC group was increased compared with that in the control group (*P* < .0001). Plasma FEZF1-AS1 could distinguish patients with NSCLC from healthy individuals via the area under the ROC curve (AUC) of 0.855 (95% CI = 0.800–0.909; *P* = .000). FEZF1-AS1 combined with neuron-specific enolase increased the area under the (ROC) curve to 0.932 (95% CI = 0.897–0.968; *P* = .018). A high expression level of plasma FEZF1-AS1 was associated with some clinical features of NSCLC. Increased expression of FEZF1-AS1 greatly improved the risk of NSCLC (adjusted OR = 2.42; 95% CI = 1.23–4.76). A significant concentration–dependent relationship was noted between risk of NSCLC and higher FEZF1-AS1 expression (*P* for trend <.001).

Plasma FEZF1-AS1 could potentially be used as a biomarker for NSCLC diagnosis.

## 1 Introduction

Lung cancer is the most common cause of cancer mortality worldwide, with non-small-cell lung carcinoma (NSCLC) constituting 85% of all lung cancers.^[1]^ In recent years, there has been a sustained progression in therapeutic strategies for NSCLC, but 5-year survival rates remain at less than 15%. Commonly NSCLCs are discovered in the later stages, leading to low survival rates. Conversely, patients who were diagnosed during the early stages of NSCLC have higher survival rates. However, an absence, or only mild typical signs of NSCLC appear until the cancer is advanced, leading to great difficulties in early diagnosis of NSCLC. Therefore, it is of significant clinical importance to screen new biomarkers for early diagnosis of NSCLC, with a need for high sensitivity and specificity.

Long non-coding RNAs (lncRNAs) are not translated into proteins, with coding transcripts over 200 nucleotides in length.^[2]^ They participate in tumorigenic, oncogenic, or tumor suppressive pathways by adjusting gene expression at the posttranscriptional and transcriptional levels. Increasing evidence demonstrates that plasma lncRNAs have the potential capacity to be used as biomarkers in various tumor diagnoses.^[3,4]^ A recently discovered lncRNA, FEZ family zinc finger 1 antisense RNA 1 (FEZF1-AS1) was located at 7q.31.32. It was reported that the expression level of FEZF1-AS1 was high in NSCLC tissue, and increased expression levels were associated with poor differentiation grade, lymph node metastasis, and advanced TNM stage. Furthermore, function experiments revealed that a higher expression of FEZF1-AS1 leads to increased cell proliferation, migration, and invasiveness.^[5]^ These previous studies indicate plasma FEZF1-AS1 as a potential novel biomarker for NSCLC diagnosis. Furthermore, we determined the expression of plasma FEZF1-AS1 in patients with NSCLC to analyze its diagnostic value in practice.

## 2 Materials and methods

### 2.1 Ethical statement and sampling

We gained the permission from the Institutional Ethics Committees of Fourth Hospital attached to Hebei Medical University (No.2019MEC100) before the plasma was collected. The clinical data of 126 NSCLC patients (57 men and 69 women, mean age: 59.381 ± 8.737 years) and 62 healthy controls (30 men and 32 women, mean age: 57.89 ± 11.34 years) were obtained in the Fourth Hospital of Hebei Medical University (Shijiazhuang, China) from December 2018 to July 2019. Whole blood was drawn into K2-EDTA tubes before chemotherapy or surgery. The samples of whole blood (5 mL) were centrifuged at 1200 × g for 20 minutes at 4°C within 30 minutes of collection. Blood plasma was then immediately gathered in RNase/DNase-free tubes, and stored at −80°C.

### 2.2 Total RNA isolation

Before RNA isolation, a *Caenorhabditis elegans* cel-miR-39 (Servicebio Co. Ltd, Wuhan, China) was spiked into the plasma samples at a final concentration of 0.2 nM and used as an external reference. RNA extracting fluid (Servicebio Co. Ltd, Wuhan, China) was used to isolate total RNA from plasma following the product description, and each sample was eluted into 15 μL of nuclease-free water. Absorbances at 260/280 nm (RNA/DNA) and 260/230 nm (RNA/protein) were detected by the NanoDrop 2000 spectrophotometer (Thermo Fisher Scientific, Waltham, MA, USA). All RNA samples were stored at −80°C.

### 2.3 Quantitative real-time polymerase chain reaction (qRT-PCR)

Total RNA was reverse transcribed into cDNA using RevertAid First Strand cDNA Synthesis Kit (ThermoFisher Scientific, Waltham, MA, USA). For reverse transcription, FEZF1-AS1 gene-specific primers and cel-miR-39 RT primers were added during cDNA synthesis. Then, the cDNA samples underwent qRT-PCR using FastStart Universal SYBR Green Master (Rox; Roche, Switzerland). qPCR cycling conditions are as follows: primary denaturation at 95°C for 10 minutes, 40 15-second cycles at 95°C, then 60°C for 60 seconds. The level of FEZF1-AS1 expression was normalized against that of cel-miR-39 by 2^-ΔΔCt^.^[6]^ ΔCt_target_ = Ct (FEZF1-AS1, sample), −Ct (cel-miR-39, sample). ΔCt_contol_ = Ct (FEZF1-AS1, control), −Ct (cel-miR-39, control). ΔΔCt = ΔCt_target_–ΔCt_control_.

Primer sequences are as follows: FEZF1-AS1: 5′-CAGTTTATGATTGCCGTCCTCC-3′, reverse, 5′-GTGATCGTCTAGGTGGTAACC-3′, cel-miR-39-S: 5′-ACACTCCAGCTGGGTCACCGGGTGTAAATC-3′, Universal Primer A: 5′-TGGTGTCGTGGAGTCG-3′, cel-miR-39-3p-RT: 5′- CTCAACTGGTGTCGTGGAGTCGGCAATTCAGTTGAG CAAGCTGA-3′.

Each experiment was performed in triplicate.

The concentrations of plasma CYFRA 21-1, neuron-specific enolase (NSE), and carcinoembryonic antigen (CEA) were detected in a Roche Cobas e601 (Roche, Switzerland) via the chemiluminescence method (cutoff values were 3.3, 4.6 ng/ml, and 15.2 μg/ml respectively).

### 2.4 Statistical analysis

GraphPad Prism 8.0.2 and SPSS 22.0 were used to generate and analyze all graphs and data, respectively. The differences between NSCLC and controls were evaluated by the Mann–Whitney *U* test. The association between plasma FEZF1-AS1 and clinicopathological features of NSCLC were analyzed by Pearson Chi-squared test and Mann–Whitney *U* test. The coefficients of FEZF1-AS1, CYFRA 21-1, NSE, and CEA were analyzed via logistic regression model. The natural logarithmic concentrations of biomarkers were considered as predictor variables. The diagnostic values of linear predictor, or logit, resulting from the multivariable model were calculated using the ROC curves. The risk of NSCLC was analyzed by multivariate analysis using nonconditional logistic regression analysis. *P* values of <.05 were considered as statistically significant.

## 3 Results

### 3.1 Plasma FEZF1-AS1 expression level in NSCLC

**Figure 1:**
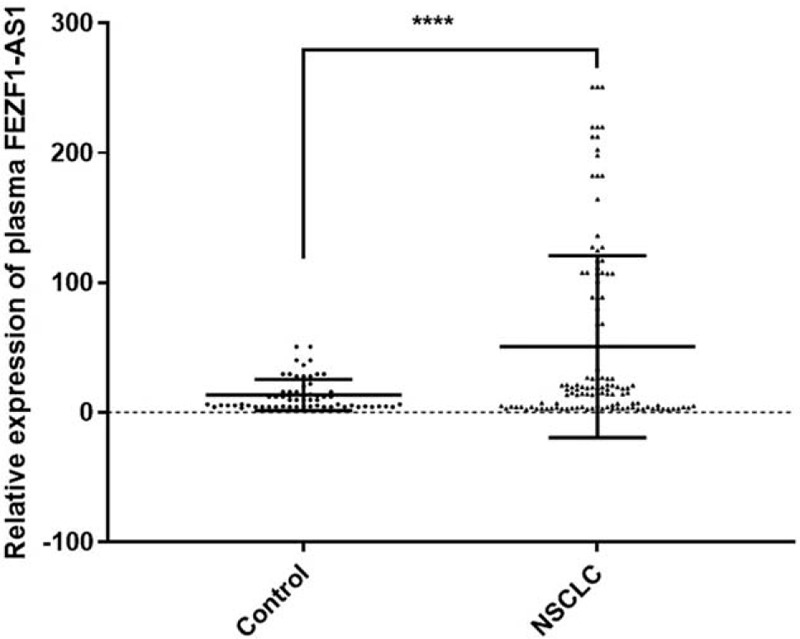
The plasma FEZF1-AS1 expression levels. QRT-PCR was used to detect plasma FEZF1-AS1 expression in NSCLC patients (n = 126) and controls (n = 62). ^∗∗∗∗^*P* < .0001.

qRT-PCR was used to detect FEZF1-AS1 expression in 126 NSCLC patients and 62 controls. To reduce variation, outliers were excluded according to the Pauta criterion. The Mann–Whitney *U* test indicated that FEZF1-AS1 expression was significantly upregulated in NSCLC patients compared with that in healthy controls (*P* < .0001; Fig. 1).

### 3.2 Diagnostic value of FEZF1-AS1 in NSCLC

**Figure 2:**
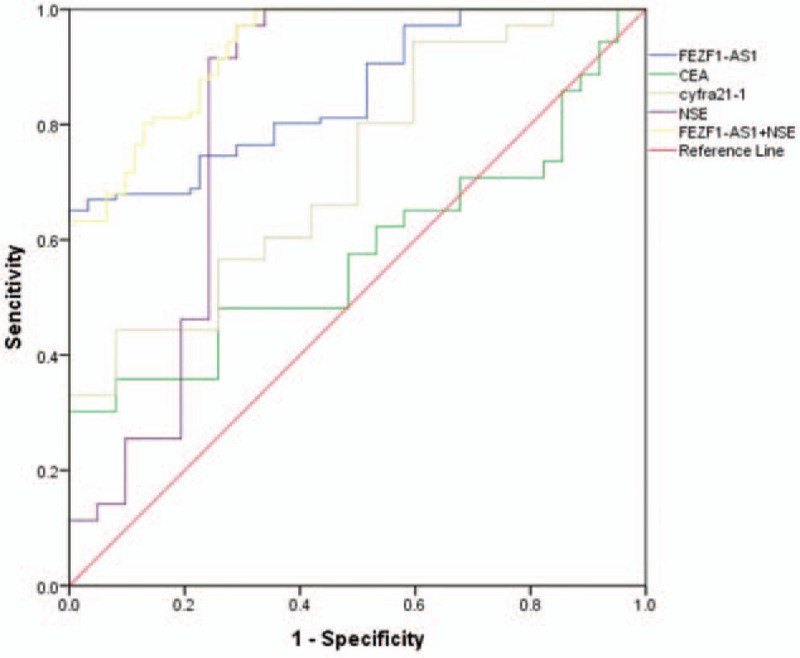
The abilities of FEZF1-AS1, NSE, FEZF1-AS1 and NSE combination, cyfra21-1, and CEA in NSCLC diagnosis.

Detection of FEZF1-AS1 expression in the blood plasma of 126 patients with NSCLC and 62 healthy controls, was analyzed via an ROC curve. The area under the (ROC) curve (AUC) was 0.855 (95% CI = 0.800–0.909; Fig. 2), indicating that the diagnostic accuracy of plasma FEZF1-AS1 expression is moderate. Nowadays, plasma CYFRA 21-1, NSE, and CEA are used to screen for NSCLC. Hence, this study compared the diagnostic capability of FEZF1-AS1 against CYFRA 21-1, NSE, and CEA. The concentrations of CEA, CYFRA 21-1, and NSE were evaluated with the same plasma. The AUC values of CYFRA 21-1, CEA, and NSE were 0.722 (95% CI = 0.645–0.799), 0.579 (95% CI = 0.494–0.664), and 0.812 (95% CI = 0.729–0.895), respectively (Fig. 2). The diagnostic capability of FEZF1-AS1 and NSE in combination was then calculated by binary logistic regression. The results showed that the diagnostic ability of FEZF1-AS1 and NSE in combination was markedly increased compared to either FEZF1-AS1 or NSE alone (AUC = 0.932, 95% CI = 0.897–0.968; Fig. 2). Specifically, the combination of FEZF1-AS1 and NSE increased the sensitivity, specificity, and accuracy of distinguishing patients with NSCLC from healthy individuals, compared with FEZF1-AS1 and NSE alone (Table 1).

**Table 1:**
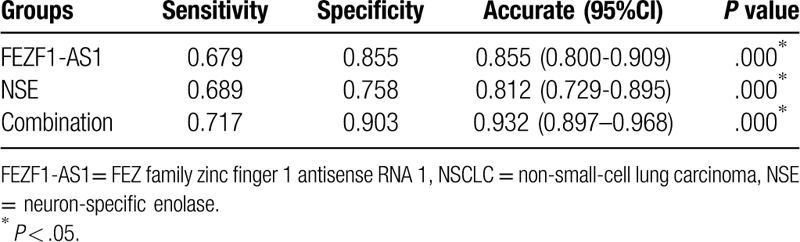
The NSCLC diagnosis abilities of the groups.

### 3.3 Association between FEZF1-AS1 expression and clinical features of NSCLC

**Figure 3:**
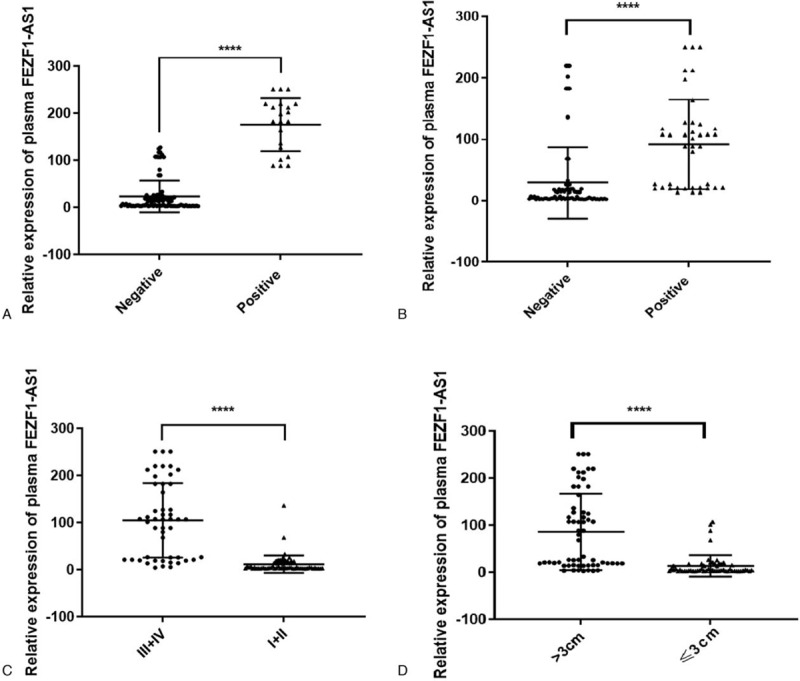
The correlations between plasma FEZF1-AS1 expression and clinical characteristics. (A) distant metastasis, (B) lymph node metastasis, (C) clinical stage, and (D) tumor size group. ^∗∗∗∗^*P* < .0001.

Patients with NSCLC were separated into high FEZF1-AS1 level (n = 65) and low FEZF1-AS1 level (n = 61) groups. The cut-off value was determined by the maximum Youden index from the ROC curves. Pearson Chi-squared test was performed to evaluate the clinicopathological parameters between the 2 groups. The correlation between plasma FEZF1-AS1 expression levels and clinicopathological features of age, gender, smoking status, histological classification, tumor size, lymph node status and metastasis, and clinical stages are summarized in Table 2. These results indicate that increased expression of plasma FEZF1-AS1 was strongly associated with male gender (*P* = .018, Table 2), positive smoking status (*P* ≤ .001, Table 2), advanced clinical stage (*P* ≤ .001, Table 2), positive distant metastasis (*P* ≤ .001, Table 2), positive lymph node metastasis (*P* ≤ .001, Table 2), and larger tumor size (*P* ≤ .001; Table 2). However, the association between high plasma FEZF1-AS1 expression against age or histological classification were not statistically significant (*P* > .05). The results indicated that plasma FEZF1-AS1 expression was significantly upregulated in patient groups with positive distant metastasis (*P* < .0001; Fig. 3A), positive lymph node metastasis (*P* < .0001; Fig. 3B), advanced clinical stage (*P* < .0001; Fig. 3C), and larger tumor size (*P* < .0001; Fig. 3D) compared to the control groups. Above all, the upregulation of plasma FEZF1-AS1 expression was seen to be correlated with NSCLC disease status.

**Table 2:**
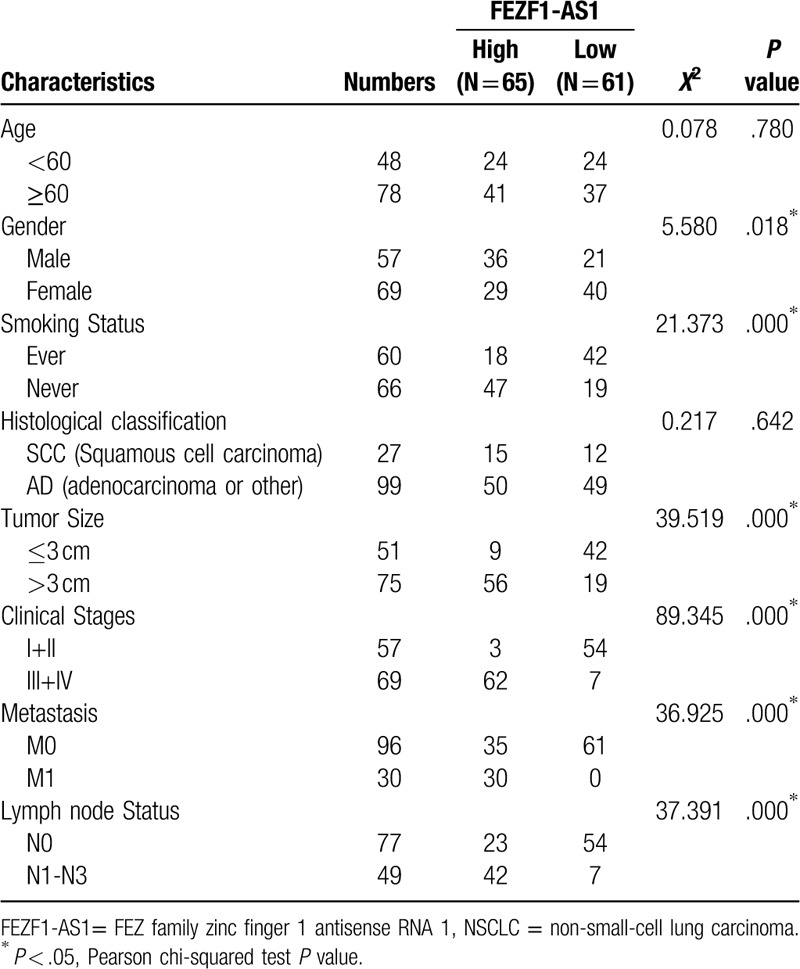
Correlation between FEZF1-AS1 and clinical characteristics in all patients with NSCLC.

### 3.4 Correlation between FEZF1-AS1 and risk of NSCLC

In this study, the risk of NSCLC as estimated by plasma FEZF1-AS1 level was analyzed by multivariate analysis via non-conditional logistic regression analysis (Table 3). The NSCLC group was divided into high- and low-FEZF1-AS1 level groups, with the median of FEZF1-AS1 expression in the control group. After adjusting for age, gender, smoking status, and NSE expression, results showed that the risk of NSCLC in the high-FEZF1-AS1 level group was notably increased (adjusted OR = 2.42; 95% CI = 1.23–4.76). We again divided the NSCLC cohort into three groups according to the tertile of FEZF1-AS1 expression in the control group. We found a significant concentration-dependent relationship between plasma FEZF1-AS1 expression level and risk of NSCLC through linear by linear association (*P* for trend <.001). Compared with the first third, the adjusted OR for the middle third is 1.93 (95% CI = 0.86–4.34) and the final third is 3.82 (95% CI = 1.72–8.47).

**Table 3:**
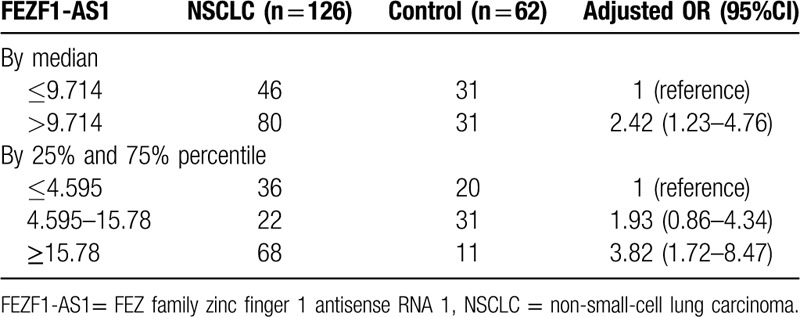
Risk of NSCLC as estimated by plasma lncRNA FEZF1-AS1.

## 4 Discussion

Among malignant cancers, lung cancer is the leading cause of death in China. Moreover, a previous study illustrated that NSCLC cells grow and divide rapidly, and their diffusion and metastasis occur at a relatively early stage; therefore, the pathogenesis of NSCLC is unclear.^[7]^ Therefore, it is important to find novel noninvasive biomarkers, which can detect NSCLC accurately and effectively at the early stages of cancer progression. Nowadays, lncRNA is recognized as a pervading factor that participates in cell proliferation, differentiation, apoptosis, and carcinogenesis.^[8]^ For example, AFAP1-AS1 indicates a poor prognosis and regulates NSCLC cells by repressing p21 expression, and may serve as a therapeutic target for human NSCLC.^[9]^ Furthermore, lncRNAs have therapeutic potential, strategies are being developed based on molecules that are designed to disrupt the expression or functionality of these lncRNAs.^[10]^

LncRNA FEZF1-AS1, a newly identified lncRNA, has been suggested as a potential target for NSCLC therapy.^[5,11]^ In addition, prior studies have noted the importance of FEZF1-AS1 in promoting tumor proliferation and metastasis in colorectal, gastric, and hepatocellular carcinoma.^[12–14]^ It was reported that FEZF1-AS1 inhibited p21 expression in gastric cancer to promote cell proliferation by LSD1-mediated H3K4me2 demethylation.^[15]^ Additionally, an lncRNA FEZF1-AS1 knockdown inhibited hepatocellular carcinoma cell migration and invasion through suppression of JAK2/STAT3 signaling-mediated epithelial-mesenchymal transition.^[16]^ Above all, these findings reveal FEZF1-AS1 as a potential diagnostic and predictive biomarker for NSCLC, however, no previous study has reported the use of plasma FEZF1-AS1 as a biomarker for NSCLC diagnosis. In this study, plasma FEZF1-AS1 was detected using qRT-PCR, and FEZF1-AS1 was found to be upregulated in patients with NSCLC, compared to the control group. Surprisingly, the combination of FEZF1-AS1 and NSE was found to have a notably higher diagnostic accuracy for detection of NSCLC compared to that of FEZF1-AS1 or NSE alone. According to previous studies, 11% to 30% cases of NSCLC demonstrated expression of neuroendocrine markers despite the absence of neuroendocrine tumor morphological traits.^[17–18]^ Preliminary studies proved that elevated NSE levels was important to discriminate NSCLC and associated with an enhanced drug sensitivity, a more aggressive clinical tumor behavior and larger tumor size.^[19–22]^ In the study, higher plasma FEZF1-AS1 level was associated with larger tumor size and poor prognosis, which was in line with the biological significance of NSE. FEZF1-AS1 and NSE combination could increase the sensitivity and specificity compared with FEZF1-AS1 or NSE alone. Thus, we infer that lncRNA combined with traditional tumor markers could increase NSCLC diagnostic efficiency. Furthermore, we evaluated the association between plasma FEZF1-AS1 expression and clinicopathological features of NSCLC, and discovered that high plasma FEZF1-AS1 levels were strongly associated with male gender (*P* = .018), positive smoking status (*P* ≤ .001), advanced clinical stage (*P* ≤ .001), positive distant metastasis (*P* ≤ .001), positive lymph node metastasis (*P* ≤ .001), and larger tumor size (*P* ≤ .001). The results are in accordance with previous studies on other lncRNAs in NSCLC.^[23,24]^ To the best of our knowledge, we are the first to report that upregulated FEZF1-AS1 expression is correlated with a high risk of NSCLC, and found a significant concentration-dependent relationship between the plasma FEZF1-AS1 level and the risk of NSCLC.

Despite these promising results, further structural research is required to explore the role of FEZF1-AS1 in the aggression and carcinogenesis of NSCLC. Furthermore, functional experiments are needed to explain the mechanism underlying the poor clinical prognosis of NSCLC.

## 5 Conclusions

The study confirmed that plasma FEZF1-AS1 expression level was clearly related to NSCLC, and the combination of FEZF1-AS1 and NSE markedly increased the diagnostic accuracy of NSCLC detection. We concluded that FEZF1-AS1 was proven a useful diagnostic biomarker, and potential predictive biomarker for NSCLC screening.

## Acknowledgments

All authors are grateful to the participated person and their family members. The paper was edited by Elsevier Language Editing Services.

## Author contributions

Yajie Huang and Guangjie Liu selected the patient samples and wrote the manuscript. Handie Ma generated the figures, Yanpeng Tian analyzed the data, Changjie Huang, Fang Liu and Yuxuan Jia made the experiments, Da Jiang funded the study. All authors read and approved the final manuscript.
